# It Takes a System: Magnesium Sulfate for Prevention of Eclampsia in a Resource-Limited Community Setting

**DOI:** 10.9745/GHSP-D-19-00261

**Published:** 2019-09-23

**Authors:** Robert L. Goldenberg, Elizabeth M. McClure

**Affiliations:** a Department of Obstetrics and Gynecology, Columbia University, New York, NY, USA.; b Research Triangle Institute, Durham, NC, USA.

## Abstract

Magnesium sulfate is not a silver bullet to reduce maternal mortality associated with preeclampsia/eclampsia. We believe a well-functioning health care system, especially at the hospital level, with competent well-trained providers, adequate equipment, and medications will likely be necessary.

*See related article by* *Williams*.

One hundred years ago in high-income countries, hypertensive diseases accounted for a large proportion of adverse pregnancy outcomes including maternal mortality, stillbirth, and neonatal mortality. Hypertensive diseases include chronic hypertension, pregnancy-induced hypertension, preeclampsia, and its major complication, eclampsia. Presently, although the incidences of chronic hypertension, pregnancy-induced hypertension, and preeclampsia have not changed substantially, rates of progression of preeclampsia to eclampsia and the maternal mortality associated with preeclampsia and eclampsia have changed.^1^ The vast majority of hypertension-related maternal mortality is associated with eclampsia, but most hypertension-related stillbirths occur in the presence of preeclampsia.^2^

One hundred years ago in high-income countries, eclampsia occurred in about 70 per 10,000 pregnancies, and the maternal case fatality rate in women with eclampsia was about 20% to 40%.^2^^–^^5^ Today, again in high-income countries, eclampsia occurs in less than 1 in 10,000 pregnancies, and the case fatality rate among women with eclampsia is less than 1%. Thus, the improvements occurred both in the progression of preeclampsia to eclampsia and the case fatality rates of women who developed eclampsia, with both complications reduced by about 99%. Most of the reductions in maternal mortality occurred between 1930 and 1970. During those years, substantial advances occurred in the increased use of antenatal care with more visits late in pregnancy, better screening for hypertension and proteinuria during those visits, increased hospitalization for women with preeclampsia, and in those hospitals, more rapid delivery for women with preeclampsia and eclampsia.^2^

## MAGNESIUM SULFATE AND REDUCTIONS IN MATERNAL MORTALITY

Appreciating that much of the preeclampsia-related mortality is associated with the development of seizures, much attention has been directed at seizure prevention.^6^ Over the years, the use of many therapeutic agents has been described including morphine and valium as well as decreased stimulation including bed rest in darkened rooms. The use of magnesium sulfate for seizure prevention was first described in 1906, and in subsequent years, its use was advocated and sometimes adopted in the United States but rarely in Great Britain.^6^^,^^7^ In 2002, the results of a large 10,000-subject, multicountry, hospital-based randomized clinical trial, the Magpie Trial, showed a significant reduction from 1.9% to 1.1% in seizures in hospitalized women with preeclampsia associated with the use of magnesium sulfate.^8^ However, there were no differences in maternal or perinatal mortality between those treated with magnesium sulfate and those not treated. Since then, the use of magnesium sulfate has been widely advocated and adopted, even in settings that differ from those in the Magpie Trial.^9^ It is important to understand that during the years of the greatest reductions in the incidence of seizures and reductions in eclampsia-related deaths, magnesium sulfate was rarely used in the United States for preventing or treating eclampsia and almost never in Great Britain.^1^

The incidence of eclampsia and the associated case fatality rates in many low-income countries today approach those seen in high-income countries 100 years ago.^1^ To better understand how mortality associated with preeclampsia/eclampsia in low- and middle-income countries could be reduced, we used mathematical models to evaluate the interventions most likely to reduce maternal mortality associated with preeclampsia/eclampsia in sub-Saharan Africa.^2^ Clearly, screening for preeclampsia by blood pressure and proteinuria measurements when combined with prompt transfer to an appropriate institution and early delivery saved the most lives. Use of magnesium sulfate alone or even when combined with other interventions resulted in fewer maternal lives saved and did not reduce the risk of stillbirths.^10^

Using magnesium sulfate alone or with other interventions resulted in fewer maternal lives saved and did not reduce the risk of stillbirths.

## COMMUNITY-LED PROGRAM TO IMPROVE ECLAMPSIA CARE

The article by Williams et al. in this issue of GHSP describes a study in Bangladesh to assess an intervention to improve identification, referral, and care for women with pre-eclampsia and eclampsia at primary care-level health facilities.^11^ The article examines the intervention's focus on the use of magnesium sulfate, but also addresses issues such as the number of antenatal visits, availability of blood pressure apparatuses, the skills of health care providers to appropriately identify women with hypertensive disease, and transfer to a higher level of care. Improving hospital care for women with preeclampsia or eclampsia was not emphasized in the intervention. The study made little attempt at determining pregnancy outcomes, and instead predominantly evaluated the health workers' compliance with the protocols to screen, provide prereferral management, and refer women with preeclampsia and eclampsia.

It is difficult to assess the quality of the health system in which this study took place. However, based on the data and the descriptions available, the quality seems challenged in this environment. Although 45 clinics were initially included in the study, only 35 had sufficient data for the analyses. The data available from the health registries appeared to be unreliable and much of it seemed inaccurate or lost. Health workers' adherence to the protocol was poor. Although attendance at antenatal care was emphasized, and most women had some care, and blood pressure measurements were taken on nearly all visits that actually took place, 65% of the women received only 1 visit. The timing during the pregnancy that the blood pressure was actually measured is unclear. As the authors' noted, why more women in the community were diagnosed with severe preeclampsia compared to mild preeclampsia was not clear because published data have suggested that many more women have mild preeclampsia compared to severe preeclampsia.^11^ These results suggest measurement error or other issues in how data were collected and recorded in the health system's database. We emphasize that to understand the impact of any intervention, ensuring high-quality data is an important first step. Overall, it appears that although the Bangladesh program described in the paper by Williams et al. is attempting to improve the preeclampsia/eclampsia-related care, to date, this program has not been successfully implemented and is unlikely to reduce maternal and fetal mortality.

## WELL-FUNCTIONING HEALTH SYSTEMS REQUIRED

If the lessons from high-income countries about successfully implementing a protocol to improve care for women with preeclampsia are considered, a well-functioning health system is almost always required. Identifying women with preeclampsia is necessary to provide adequate care. This requires routine, quality antenatal care with regular visits later in pregnancy when preeclampsia is likely to become apparent. The minimum of 4 visits recommended for this program (in line with Bangladesh policy at the time of implementation) without clarifying when during pregnancy these visits should occur will likely result in inadequate care.^11^ Although the World Health Organization (WHO) initially recommended a minimum of 4 prenatal visits, in 2016, WHO increased the recommended minimal number of visits to 8, mostly to better screen for preeclampsia.^12^ In high-income countries, the recommended frequency of prenatal visits varies, but generally increases in the third trimester of pregnancy and often occurs weekly during the last month of pregnancy. This type of visit schedule was adopted in the United States to screen for preeclampsia late in pregnancy.

To diagnose preeclampsia effectively, blood pressure and proteinuria measurements are needed at each antenatal care visit. Functioning equipment, a well-trained staff, and appropriate protocol, are necessary. In the Bangladesh protocol, substantial emphasis is placed on administering a loading dose of magnesium sulfate before transfer. With the diagnosis of severe preeclampsia, after a loading dose of magnesium sulfate, transfer to a facility with the capabilities to affect an immediate delivery (i.e., availability of cesarean delivery and induction of labor 24 hours a day, 7 days a week) is recommended. From the data presented, these requirements were met only about half the time. Another issue was that, although the referral protocol indicated referral to a higher level of care for women with less severe pre-eclampsia, the study findings indicated that most of the time this protocol was not followed. The care provided for those women with less severe preeclampsia is not clear, although referral was recommended. This is an important consideration, especially because mild preeclampsia can rapidly progress to severe preeclampsia or eclampsia.^13^ Managing women with severe preeclampsia and eclampsia, or even mild preeclampsia, is far more complicated than administering magnesium sulfate alone. Without comprehensive care by well-trained clinicians, women will die unnecessarily. We note again that little emphasis in the intervention assessed in this study was placed on hospital care. We believe that the use of magnesium sulfate may be overstressed in this intervention, and the extensive focus on provision of magnesium sulfate may detract from the ability of the health center staff to focus on appropriate screening coupled with referral. We strongly believe that diagnosing preeclampsia, transfer to a facility, and early delivery should be the primary focus of preeclampsia/eclampsia improvement programs, not magnesium sulfate.

Diagnosing preeclampsia, transfer to a facility, and early delivery should be the primary focus of improvement programs, not magnesium sulfate.

Several recent studies that focused on community diagnosis and transfer of the pregnant woman with complications to a facility have failed to reduce mortality. A multicountry randomized trial, the Emergency Obstetric and Neonatal Care study, which focused on community identification of various pregnancy complications and transfer, failed to show a reduction in mortality.^14^ The First Look study, a trial focusing on ultrasound use during antenatal care and transfer for complications,^15^ also failed to reduce mortality. The results of the Community Level Interventions for Preeclampsia (CLIP) study,^16^^–^^17^ a randomized trial conducted in 4 low-and middle-income countries, specifically aimed at reducing mortality from preeclampsia/eclampsia identified in the community, failed to show mortality reductions at any of the sites. As examples, the conclusions from the India site were that “community-level interventions for pre-eclampsia did not improve maternal, fetal, or newborn mortality or major morbidity.” Likewise, the authors from the Mozambique site found, “The CLIP intervention was not associated with an improvement in the primary outcome.” And finally, from the Pakistani site, the investigators noted, “Overall, the CLIP trial did not show significant impact on the primary composite maternal and perinatal death or morbidity endpoint.” Together, these studies suggest that without adequate hospital care, mortality reductions may not occur, regardless of what community-level interventions are introduced. Simple interventions introduced at the hospital level have also failed to reduce mortality, as a study on checklists within hospitals in India also failed to show benefit.^18^

Therefore, we believe these results indicate that even diagnosing pregnancy-related complications in the community with transfer is not sufficient to achieve substantial reductions in mortality, if the hospital care is inadequate. Taking care of women, fetuses, and newborns with complicated medical conditions is difficult and often requires substantial provider training, good judgment, and excellent medical and sometimes surgical skills. Brief trainings of health workers will likely not accomplish the goal of substantial reductions in mortality. Although we applaud the effort to address a serious health issue in Bangladesh, the introduction of “silver bullets” such as magnesium sulfate, used alone or even with other interventions, will also not likely achieve reductions in mortality. Instead, a well-functioning health care system staffed with competent well-trained providers with adequate equipment and medications will likely be necessary to provide appropriate care to women with preeclampsia/eclampsia, as well as those with other complicated conditions, and reduce the associated mortality.

## Notes

**First Published Online:** September 16, 2019
